# Building an eye care team in rural areas: a central Indian case study

**Published:** 2018-07-31

**Authors:** Elesh Jain, Subeesh Kuyyadiyil

**Affiliations:** 1 Consultant: Paediatric Ophthalmology and Hospital Administrator, Sadguru Netra Chikitsalaya; 2 Assistant Administrator: Sadguru Netra Chikitsalaya

**If attracting, retaining and motivating eye care professionals to give their best is challenge in cities it is a seemingly impossible task in remote rural areas. Sadguru Nethra Chikitsalaya (SNC) at Chitrakoot is one of the few hospitals that have turned its rural setting to its advantage. Strategies and details described in this article will help countless other institutions that are struggling with similar issues.**

**Figure:**
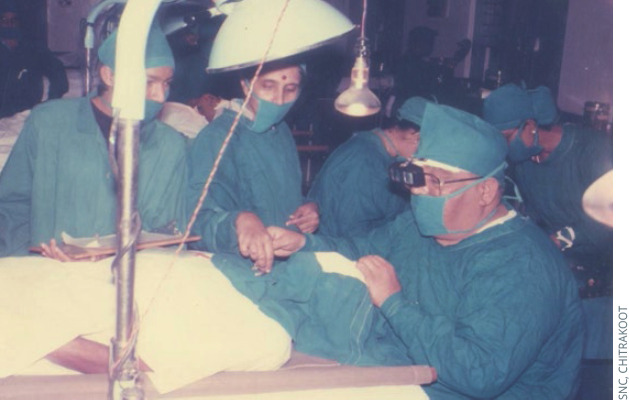
One of the surgical camps at Chitrakoot. INDIA

Chitrakoot is an important Hindu pilgrimage centre in the state of Madhya Pradesh, India. All throughout the year, thousands of pilgrims throng this small town from various parts of India. Apart from the religious significance of this town, Chitrakoot is also famous for its quality eye care services. From only a few camps a year during winter many years ago, to a state-of-the-art eye hospital, Chitrakoot has come a long way.

Shri Sadguru Seva Sangh Trust engaged in eye care delivery to the socially neglected communities in Chitrakoot since the 1950s. The organisation had conducted occasional eye camps, especially during winters, as patient turnout was huge compared to other seasons of the year. With the patronage of the founder of the Trust, Ranchhoddasji Maharaj, such programmes managed to serve people even in Chtirakoot's challenging terrain.

Surgical services were mainly organised and provided by voluntary ophthalmologists and nurses from various parts of India, while local volunteers were also involved. Huge tents were laid out to perform eye surgeries and facilitate pre- and post-operative care. Seeing that the annual camps had a huge turnout and a large number of beneficiaries, the Trust management decided to extend the services around the year and established a full-fledged eye hospital in the year 2000, named Sadguru Netra Chikitsalaya (SNC).

With the infrastructure in place, ensuring availability of human resources for provision of services throughout the year was a key challenge. Chitrakoot is a rural area and lacks proper connections with other major towns, good educational institutions for children or even a social life. These were significant barriers for hiring and retaining people. Surgeries were limited to the winter season, and so ophthalmologists were reluctant to join the organisation for a long-term career or training. As a result, till 2002, almost 98% of the workload was taken up during the winter months with support from volunteers.

## The value of leadership

Dr B K Jain, an ophthalmologist and an ardent supporter of the Trust's mission, joined the eye care division in 1973. Dr Jain was newly married and had to face several challenges convincing his wife to shift from a cosmopolitan city like Mumbai to rural Chitrakoot. However, his passion for service drove Dr Jain to survive and build the organisation. His unique style of leadership ensured others in the team felt valued and brought together people from diverse backgrounds.

## Challenges

The Trust was unable to afford permanent staff as there were almost no work during summers. This led to concerns around plans for further expansion of services. As a majority of surgeries were done without IOL implants, quality of the cataract surgery was a big concern. Surgical follow-up among the patients was also poor. Financial constraints added to the list of challenges as the funds came entirely from donors. It limited the hospital's ability to provide adequate financial remuneration and amenities to employees. This even made it difficult to afford state-of-the-art facilities and technical advancements for the patients.

## Change

To tackle the seasonal imbalance in patient inflow, the organisation initiated many community-based approaches including cataract screening camps and the establishment of vision centers in remote areas. Collaborations with several eye care hospitals such as Aravind Eye Care System, non-governmental organisations (NGOs) and iNGOs such as SEVA and Orbis provided the exposure and expertise to add speciality services.

To assure quality in cataract surgical services, a policy of ‘intraocular lenses (IOL) for everyone’ was implemented with capacity improvement. With the establishment of the School of Paramedical Science at Chitrakoot in the year 1999, the Trust ensured availability of qualified and trained allied ophthalmic staff to the organisation. This was a huge opportunity for ophthalmologists to enhance their productivity without compromising on quality.

To cope with rising financial requirements, in the year 2002 the Trust created a fully paid and a subsidised wing for patients who could afford to pay for the services. The Trust offered the choice of opting for free, subsidised or paid services to the patient themselves. As the patient inflow to paid and subsidised segments increased, it helped to provide adequate financial benefits and better living conditions for the employees on the campus. Professional growth of ophthalmologists and other essential cadres was ensured by offering them tiered training courses for all cadres of eye care staff. Continuing medical education (CME) sessions and workshops with visiting faculty helped in keeping the knowledge current and improved the prestige of the hospital.

**Figure:**
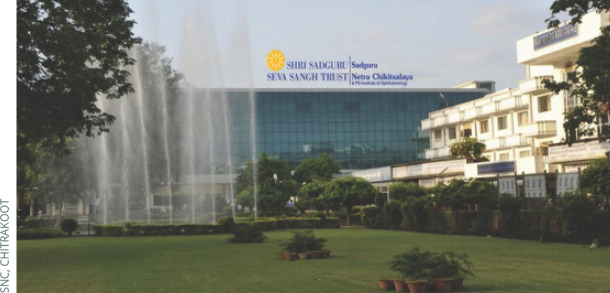
State of the art eye hospital at Chitrakoot. INDIA

An overhaul of the information technology (IT) infrastructure was done to improve data management and global connectivity. A dedicated broadband connection made it possible to include tele-ophthalmology at SNC and also considerably improved communication and entertainment for the employees.

Provision of good quality living quarters, shopping facilities, an English-medium school for children and strengthening the *Mahila* (women's) wing for gainful employment of wives of employees were some of the measures taken by the management that were helpful in retention of employees in the long run. These strategies not only ensured an increase in retention of ophthalmologists, but also helped in the establishment of super-speciality departments including a paediatric eye care centre and a vitreo-retinal department at Chitrakoot.

## Progress

Today, SNC is one of the largest rural eye care providers In India with more than 85 ophthalmologists working round the year with the support from 600 para-clinical and support staff. It is also considered as one of the pioneer institutes in the field of community ophthalmology. Its successful engagement with the community to tackle seasonal imbalances is well recognised resulting in more than 35,000 surgeries in summer months. As an organisation committed to its community, today SNC helps various other eye hospitals to tackle such issues and improve overall performance through a continued consultancy programme.

The cataract surgical volume grew year on year with 100% IOL implants, and today it is one of the few organisations in the world to perform more than 100,000 cataract surgeries each year. The establishment of 40 primary eye care centres (vision centres) spread across Madhya Pradesh and Uttar Pradesh ensures an increased cataract follow-up rate.

With increased retention of ophthalmologists, improved systems and quality assurance, the hospital today is able to provide comprehensive eye care services in appreciable volumes.

The institute is also a recognised centre for training government surgeons and ophthalmic assistants from various parts of India. A large number of private organisations within India and other countries also enrol for the post-graduate training programme. Affiliation with the International Council of Ophthalmology (ICO), has so far enabled ophthalmologists from 12 countries enrol into different programmes. The paramedical wing also provides quality training to several rural youth through its structured courses that include diplomas in ophthalmic assistance, vision technicians, health care workers, operation theatre assistants and lab assistants.

To cope with the high volume and ensure quality, modern infrastructure and equipment were added including 25 state-of-the art modular operation theatres and a world-class central sterile supply department (CSSD). These advancements help us perform 600- 800 surgeries each day.

**Table 1:**
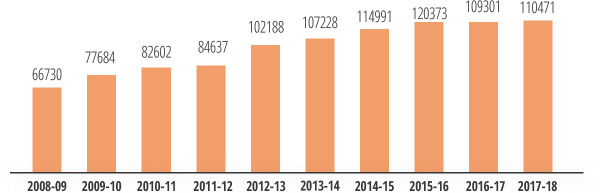
Cataract surgeries from 2008- 09 to 2017-18

## Key Learnings

- Leadership with long-term vision is the key to bringing people together in difficult rural areas.
- The value-based transparent system gives people the confidence to be a part of the system, tackle difficulties and grow together.
- Continuous focus on improvement coupled with partnerships with similar organisations can help build sustainable systems with high volume, quality and increased financial viability.
- Training programmes promote a continued inflow of aspiring workforce and improve the overall functioning and quality.
- Involving employees in decision making for continuous improvement builds a team that is effective and efficient.
- An encouraging work environment and extending support for social life can enhance overall contribution and loyalty of employees towards the organisation.

**Table 2:** Speciality surgeries 2017-18

Speciality	Total number of surgeries
**Glaucoma**	4,032
**Vitreo-retina**	6,067
**Cornea**	4,203
**Orbit and oculoplasty**	5,780
**Strabismus**	303
**Refractive surgery**	745
**Paediatric surgeries (squint & others)**	3,193

